# Heart Rate Variability in Head-Up Tilt Tests in Adolescent Postural Tachycardia Syndrome Patients

**DOI:** 10.3389/fnins.2020.00725

**Published:** 2020-08-11

**Authors:** Maija Orjatsalo, Anniina Alakuijala, Markku Partinen

**Affiliations:** ^1^Department of Clinical Neurophysiology, HUS Medical Imaging Center, Helsinki University Hospital, Helsinki, Finland; ^2^Department of Neurological Sciences, University of Helsinki, Helsinki, Finland; ^3^Vitalmed Helsinki Sleep Clinic, Helsinki, Finland

**Keywords:** postural tachycardia syndrome, adolescent, head-up tilt test, autonomic nervous system, heart rate variability

## Abstract

**Introduction:** Postural tachycardia syndrome (POTS) is a suspected dysautonomia with symptoms of orthostatic intolerance and abnormally increased heart rate while standing. We aimed to study cardiac autonomic nervous system functioning in head-up tilt (HUT) in adolescents with POTS to find out if parasympathetic tone is attenuated in the upright position.

**Methods:** We compared characteristics of a group of 25 (females 14/25; 56%) adolescents with POTS and 12 (females 4/12; 34%) without POTS aged 9–17 years. We compared heart rate variability with high- and low-frequency oscillations, and their temporal changes in HUT.

**Results:** The high-frequency oscillations, i.e., HF, attenuated in both groups during HUT (*p* < 0.05), but the attenuation was bigger in POTS (*p* = 0.04). In the beginning of HUT, low-frequency oscillations, i.e., LF, increased more in POTS (*p* = 0.01), but in the end of HUT, an attenuation in LF was seen in the POTS group (*p* < 0.05), but not in the subjects without POTS. There were no associations of previous infections or vaccinations with POTS. Subjects with POTS were sleepier and their overall quality of life was very low.

**Conclusion:** The results imply to an impaired autonomic regulation while standing in POTS, presenting as a lower HF and higher LF in the beginning of HUT and an attenuated LF in the prolonged standing position.

## Introduction

Postural tachycardia syndrome (POTS) is a type of functional dysautonomia characterized by symptoms of orthostatic intolerance accompanied with abnormally increased heart rate (HR) while standing (Sheldon et al., 2015). Diagnostic criteria for POTS are symptoms of orthostatic intolerance lasting ≥ 6 months together with an increase in HR of ≥ 30 beats per minute (bpm) for at least 30 s when moving from a recumbent to a standing position but without simultaneous orthostatic hypotension [>20 mmHg drop in systolic blood pressure (SBP)], and other causative conditions or medications must be ruled out (Sheldon et al., 2015). For subjects under 19 years old, an increase of HR ≥ 40 bpm and symptoms of orthostatic intolerance are needed (Sheldon et al., 2015).

A prevalence of 0.2% with a 4:1–5:1 female/male ratio has been described for POTS (Kimpinski et al., 2012; Sheldon et al., 2015). The number of POTS diagnoses is increasing, presumably as a result of better disease recognition (Skufca et al., 2017; Brinth et al., 2018).

In addition to symptoms of orthostatic intolerance, POTS patients often suffer from chronic pain, gastrointestinal symptoms, fatigue, and sleep disturbance, and symptoms worsen with upright position (Sheldon et al., 2015). Patients with POTS have only mild symptoms of general autonomic failure (Kimpinski et al., 2012), and orthostatic intolerance is the most severe autonomic symptom.

The cause of POTS has not yet been established (Benarroch, 2012), though many possible disease mechanisms including autonomic denervation (Kimpinski et al., 2012; Haensch et al., 2014), autonomic autoimmunity (Ruzieh et al., 2017), hypovolemia (Benarroch, 2012; Kimpinski et al., 2012), hyperadrenergic stimulation (Kimpinski et al., 2012), and deconditioning have been proposed (Benarroch, 2012; Fu and Levine, 2015). The entity of symptoms in POTS patients is likely caused by a combination of these factors.

Heart rate variability (HRV) is an ECG-based method to assess cardiac autonomic nervous system (ANS) (Camm et al., 1996). HR has a constant beat-to-beat temporal variation mediated by autonomic sympathetic and parasympathetic nervous system influences on the sinus node. HRV is a good method to estimate the parasympathetic nervous system (high-frequency oscillations, HF), but the method is not so well validated in measuring the sympathetic nervous system, and low-frequency oscillations, LF, consist of both parasympathetic and sympathetic influences on the sinus node (Camm et al., 1996). LF/HF depicts the ratio of LF and HF (Camm et al., 1996).

In recent years, numerous studies of POTS patients’ head-up tilt (HUT) test describing HRV have been published (Furlan et al., 1998; Corkal et al., 2014; Baker et al., 2018a; Swai et al., 2019), but only a few with adolescent subjects (Stewart, 2000; Swai et al., 2019). In one study, a different definition of POTS was used, in other words, a milder heart rate increase was accepted (Yoshida et al., 2014). HRV parameters (HF and LF) during HUT test correlated with autonomic dysfunction in POTS and in other autonomic neuropathies (Baker et al., 2018b). HRV was lower for POTS patients than for controls in rest and during HUT tests (Stewart, 2000; Baker et al., 2018a; Jacob et al., 2019). Parasympathetic tone seemed to decrease in the upright position in HUT tests (Baker et al., 2018a). POTS patients showed a significant increase in LF/HF with tilt compared with the supine position (Corkal et al., 2014). Resting LF/HF in POTS was elevated and it increased more with HUT (Furlan et al., 1998). However, using direct nerve recording, resting sympathetic activity of POTS patients was found to be low (Jacob et al., 2019) or normal (Bonyhay and Freeman, 2004; Muenter et al., 2005; Lambert et al., 2008). A decrease in the cerebral blood flow of POTS patients in the HUT test was associated with an increase in the peripheral sympathetic tone (Del Pozzi et al., 2014).

In the present study, our aim was to present and compare clinical and demographic characteristics of a group of adolescent POTS patients with a group of adolescent patients without POTS in Finland. In addition, we aimed to compare HRV and autonomic functional parameters [blood pressure (BP), HF, LF, LF/HF], and their temporal changes between the subjects positive and negative for POTS in different states of HUT tests in the group level. We hypothesized that HF, ergo parasympathetic tone, would decrease more in the POTS group in the HUT test compared with the subjects without POTS.

## Materials and Methods

We systematically analyzed data obtained from all adolescent patients (years 9–17), who had been studied for suspected dysautonomia in Vitalmed Helsinki Sleep Clinic in 2015–2018. Vitalmed Helsinki Sleep Clinic is a private sleep clinic where patients from all over Finland – but predominantly from the Helsinki area – visit either self or insurance paid or are referred and paid for by their municipal health care. All the patients in this age group who had been subject to HUT test were included in the analysis. Thirty-eight adolescents fulfilled these conditions. All the adolescents tested had symptoms indicating dysautonomia – e.g., orthostatic intolerance – for which reason the tests were clinically conducted originally; thus, there were no healthy controls. In addition, we analyzed patient files and questionnaires to obtain demographic and clinical data.

One of the subjects had consumed propranolol 10 mg daily during the HUT test; thus, the subject was excluded from the analysis. This left us with 37 subjects. None of the other subjects used medication that influenced HR or BP before or at the time of the measurements.

In our analysis, we compared the groups with and without clinical POTS. We analyzed the raw data of the HUT tests individually and made group-level comparisons. The subjects included in the POTS group met the current criteria for POTS (Sheldon et al., 2015). A neurologist with experience on dysautonomia confirmed the diagnoses of POTS.

The study was conducted according to the declaration of Helsinki. Data of all the subjects were anonymized before the analysis of the data. Institutional review board of Vitalmed Helsinki Sleep Clinic approved the study. As the study was based only on documents completed during normally scheduled outpatient visits and diagnostic procedures, no written informed consent was required.

### Continuous ECG and BP Measurements

All the subjects had undergone an in-laboratory HUT test with continuous BP and ECG measurement with SOMNOscreen system (Somnomedics, Randensacker, Germany). The values of the parameters were exported using the build-in export data tool from the SOMNOscreen program. The SOMNOscreen system included beat-to-beat continuous, non-invasive BP measurement based on pulse transit time (PTT) from ECG leads to pulse oximeter at fingertip. PTT method is a validated and patented non-invasive technique to study ambulatory BP (Bilo et al., 2015). BP was individually calibrated for each subject three times in the recumbent position using a cuff method. ANS activity was analyzed using frequency domain methods of HRV (HF, LF, LF/HF) equivalent to the guidelines given by the European Society of Cardiology and the North American Society of Pacing and Electrophysiology, but 1–3 min time intervals were used (Camm et al., 1996; Berntson et al., 1997). Fast Fourier transform was used in the spectral analysis. The ECG signal was sampled at 256 Hz and was visually and automatically scanned for artifacts and non-sinus rhythms. The collected parameters were HR, high-frequency components of HRV spectra in absolute units (HF, 0.15–0.4 Hz), low-frequency components of HRV spectra in absolute units (LF, 0.04–0.15 Hz), LF/HF ratio, systolic blood pressure (SBP), diastolic blood pressure (DBP), and mean arterial pressure (MAP).

### HUT Test

All the tests were performed between 10:00 and 12:00 in a quiet room. The subjects were asked to rest in recumbent position for at least 10 min. The preferred active standing HUT method was used (Chémali and Chelimsky, 2014): the patients were asked to stand up leaning to a wall at an angle of 70 degrees from the horizontal. The HUT test lasted for 25 min unless the subject felt unbearable symptoms or symptoms of presyncope before the end of the test (9/37 patients had to stop the test before 25 min; none of their data was excluded from the analysis).

We collected the data of specific parameters – HR, HF, LF, LF/HF, DPB, SBP, and MAP – during these described time intervals in the HUT test. Pre-tilt supine: 2 min before upright position in recumbent position. HUT 1 min: during the first minute of upright position. HUT 2–4 min: from the second to fourth minutes of upright position. HUT final: during the last minute of upright position. Post-tilt supine: from the first to third minute after returning to the recumbent position. Average HUT: the average of the parameters during the whole time in the upright position.

These time intervals were selected for the analysis because we wanted to analyze the temporal variation of the ANS during the HUT test. Because the test length was different in some individuals, we did not use minute-by-minute analysis of the whole test period. We compared the individual increases and decreases of HRV, HR, and BP parameters between the groups of subjects with and without POTS. The patients were classified in the POTS group if they fulfilled criteria for POTS in the HUT test (Sheldon et al., 2015), the others were classified in the non-POTS group.

### Subjective Questionnaires

The subjects had completed questionnaires including Epworth Sleepiness Scale (ESS), Rimon’s Brief Depression Scale (RDS) (Keltikangas-Jarvinen and Rimon, 1987), World Health Organization–Five Well-Being Index (WHO-5 Wellbeing scale) (Topp et al., 2015), and Conners 3AI attention-deficit hyperactivity disorder (ADHD) screening tool (MHS, 2018). In addition, the subjects filled Visual Analog Scales for Quality of Life and General Health, ranging from 0 to 100%, 100% being the best quality and health. Subjects also filled Basic Nordic Sleep Questionnaire with questions of daytime tiredness, sleepiness, and constipation (Partinen and Gislason, 1995). Information about the use of a wheelchair, stomach pain, and limb pain was obtained from patient files. Information about confirmed microbial infections before the starting point of the symptoms were obtained from patient files. The data about previous vaccinations were obtained from a questionnaire that all the patients had filled with their parents. Patients were also asked to report the date of the Pandemrix vaccination and of the Cervarix vaccination.

### Statistical Analyses

Statistical analyses were performed using Stata 14.2 (StataCorp, TX, United States). The different variables were tested for normality using the Shapiro-Wilk test. Group comparisons between subjects with and without POTS in selected time intervals of the HUT test were conducted using the two-sample Wilcoxon rank-sum (Mann–Whitney) test, and *p*-values were computed with z scores when the variables were non-parametrically distributed, and with the independent samples *t* test when the parameters were parametrically distributed. The temporal changes of each dependent variable between different intervals were conducted using the sign test, as the distributions were non-parametrical. The results were confirmed with the dependent-samples *t*-test. A *p* < 0.05 was used to denote significance.

Similarity between the groups of subjects (POTS and non-POTS) were tested with logistic regression or with Fisher’s exact test, when appropriate.

## Results

### Comparison of Demographic and Clinical Characteristics Between the Groups

Twenty-five (68%) of the tested adolescents fulfilled the previously described criteria of POTS, and 12 did not. There were no significant differences in age, sex, or body mass index (BMI) between the groups with and without POTS. Table 1 shows the demographic and clinical characteristics and selected symptoms of the subjects with and without POTS.

**TABLE 1:** Clinical characteristics of subjects with and without POTS.

	**POTS *n* = 25**	**Non-POTS *n* = 12**	***P*-value**
Age (years)*	14 (9–17)	11 (9–16)	0.136^‡^
Female/male	14:11	4:8	0.295^†^
BMI (kg/m^2^)*	20.1 (14.7–28.2)	19.5 (14.2–29)	0.682^‡^
ESS score at least 10**	6/20 (30%)	0/11 (0%)	0.066^†^
RDS at least 12**	4/20 (20%)	1/12 (8%)	0.623^†^
WHO-5*	32 (8–96)	52 (16–88)	0.289^†^
Conners 3AI score at least 10**	8/19 (42%)	5/12 (42%)	1.000^†^
Quality of life, Visual Analog Scale*	47% (0–100%)	60% (8–100%)	0.518^‡^
General health, Visual Analog Scale*	32% (0–95%)	60% (25–100%)	0.101^‡^
Sleepiness for at least 3 days a week**	12/20 (60%)	2/12 (17%)	0.028^†^
Tiredness for at least 3 days a week**	16/20 (80%)	8/12 (67%)	0.433^†^
Constipation at least 3 days a week**	2/18 (11%)	2/12 (17%)	1.000^†^
Abdominal pain**	9/25 (36%)	3/12 (25%)	0.711^†^
Limb pain**	14/25 (56%)	5/12 (42%)	0.495^†^
Subject in a wheelchair**	4/25 (16%)	1/12 (8%)	1.000^†^

*Values are means with range. **Values are numbers of subjects with percentage. ^†^P-values were computed with Fisher’s exact test. ^‡^P-values were computed with logistic regression. POTS, subjects with postural tachycardia syndrome; non-POTS, subjects without postural tachycardia syndrome; BMI, body mass index; ESS, Epworth Sleepiness Scale; RDS, Rimon’s Brief Depression Scale; WHO-5, World Health Organization–Five Well-Being Index; Conners 3AI, Conners 3AI attention-deficit hyperactivity disorder screening tool.

There were no significant differences in the number of patients who had a score of ≥12 in RDS, which refers to at least moderate depression (Keltikangas-Jarvinen and Rimon, 1987), or Conners 3AI ADHD screening tool scores between the groups. In other words, there were no significant differences in depressive symptoms or ADHD symptoms. The overall quality of life and perceived general health were low in patients with POTS. There was a trend to lower subjective experience of general health in the POTS group, but the difference was not statistically significant. Subjects with POTS felt statistically significantly more often sleepy than subjects without clinical POTS. One of the patients in the POTS group had narcolepsy type 1. There were no differences in the occurrence of constipation or pain between the groups. Four patients in the POTS group were in a wheelchair because of severe subjective symptoms of orthostatic intolerance.

Supplementary Table S1 depicts the history of microbial infections and vaccinations before symptoms. Sixty percent of POTS patients described symptoms of antecedent infections before symptoms. None of the described viral or bacterial infections were significantly more common in the POTS group. There was a trend to *Mycoplasma pneumoniae* infections being more frequent in the POTS group but, owing to small sample size, the difference was not significant. In both groups, all the patients had undergone the National Immunization Program (NIP) in Finland. There were no significant differences in the frequency of Pandemrix (ASO3 adjuvanted AH1N1 influenza) or Cervarix [human papillomavirus (HPV)] vaccinations between the groups. The Cervarix vaccination was obtained between 2013 and 2015 and the Pandemrix vaccination 2009.

### HR, HF, LF, LF/HF, and BP in HUT Tests

Table 2 shows the average HR and SBP values in group level in the selected time intervals during the HUT and the change in HR from supine to upright (delta HR). Tables 3–5 depict the group averages of individual changes of the absolute values of the HRV parameters between selected time intervals.

**TABLE 2:** Average HR and SBP during the HUT tests.

	**POTS *n* = 25**	**Non-POTS *n* = 12**
Pre-Tilt supine HR* (beats per minute)	74.6 (10.20)	74.08 (11.10)
HUT 1 min HR* (beats per minute)	103.9 (13.15)	99.2 (13.41)
HUT 2–4 min HR* (beats per minute)	100.8 (13.38)	94.3 (11.34)
HUT final HR* (beats per minute)	108.6 (17.77)	95.3 (14.49)
Post-Tilt supine HR* (beats per minute)	74.9 (13.16)	71.1 (8.68)
Maximal heart rate upright* (beats per minute)	133.8 (12.19)	126.5 (15.10)
Delta HR** (beats per minute)	52 (40–70)	34.75 (29–39)
Pre-Tilt supine SBP* (mmHg)	108.7 (10.62)	103.9 (12.56)
Average HUT SBP* (mmHg)	111.1 (13.42)	105.7 (15.32)
Post-Tilt supine SBP* (mmHg)	111.4 (12.61)	105.5 (16.29)

*Values are means with standard deviation. **Values are means with range. POTS, subjects with postural tachycardia syndrome; non-POTS, subjects without postural tachycardia syndrome; HR, heart rate in beats per minute; SBP, systolic blood pressure in mmHg; Pre-Tilt supine, 2 min before upright position in recumbent position; HUT 1 min; during the first minute of upright position; HUT 2–4 min, from the second to fourth minutes of upright position; HUT final, during the last minute of upright position; Post-Tilt supine, from the first to third minutes after returning to the recumbent position; Average HUT, the average of the parameters during the whole time in the upright position.

**TABLE 3:** Temporal changes in HF in head-up tilt tests.

	**POTS *n* = 25**	***P*-value**	**Non-POTS *n* = 12**	***P*-value**
Change in HF from Pre-Tilt supine to HUT 1 min	463 (391) to 402 (331)	<0.0001	364(216) to 330 (193)	0.0002
Change in HF from Pre-Tilt supine to HUT 2–4 min	463 (391) to 279 (221)	<0.0001	364(216) to 248 (135)	0.0032
Change in HF from Pre-Tilt supine to Average HUT	463 (391) to 163 (119)	<0.0001	364(216) to 180 (127)	0.0032
Change in HF from Pre-Tilt supine to HUT final	463 (391) to 85 (69)	<0.0001	364(216) to 134 (160)	0.0059
Change in HF from HUT 1 min to HUT final	402 (331) to 85 (69)	<0.0001	330(193) to 134 (160)	0.0059
Change in HF from HUT final to Post-Tilt supine	85 (69) to 236 (174)	<0.0001	134(160) to 299 (145)	0.0059

*Values are means of the HRV parameters with standard deviation. P-values were computed with the sign test. POTS, subjects with postural tachycardia syndrome; non-POTS, subjects without postural tachycardia syndrome; HF, high-frequency components of heart rate variability spectra in ms^2^; Pre-Tilt supine, 2 min before upright position in recumbent position; HUT 1 min, during the first minute of upright position; HUT 2–4 min, from the second to fourth minutes of upright position; HUT final, during the last minute of upright position; Post-Tilt supine, from the first to third minutes after returning to the recumbent position; Average HUT, the average of the parameters during the whole time in the upright position.*

**TABLE 4:** Temporal changes in LF in head-up tilt tests.

	**POTS n = 25**	***P*-value**	**Non-POTS n = 12**	***P*-value**
Change in LF from Pre-Tilt supine to HUT 1 min	457 (183) to 465 (178)	0.0013	450 (171) to 464 (166)	0.0730
Change in LF from Pre-Tilt supine to HUT 2–4 min	457 (183) to 431 (159)	0.0539	450 (171) to 420 (136)	0.3872
Change in LF from Pre-Tilt supine to Average HUT	457 (183) to 363 (154)	0.0073	450 (171) to 388 (152)	0.1938
Change in LF from Pre-Tilt supine to HUT final	457 (183) to 306 (183)	0.0073	450 (171) to 379 (190)	0.1133
Change in LF from HUT 1 min to HUT final	465 (178) to 306 (183)	0.0005	464 (166) to 379 (190)	0.1133
Change in LF from HUT final to Post-Tilt supine	306 (183) to 379 (209)	0.0001	379 (190) to 432 (148)	0.1133

*Values are means of the HRV parameters with standard deviation. P-values were computed with the sign test; POTS, subjects with postural tachycardia syndrome; non-POTS, subjects without postural tachycardia syndrome; LF, low-frequency components of heart rate variability spectra in ms^2;^ Pre-Tilt supine, 2 min before upright position in recumbent position; HUT 1 min, during the first minute of upright position; HUT 2–4 min, from the second to fourth minutes of upright position; HUT final, during the last minute of upright position; Post-Tilt supine, from the first to third minutes after returning to the recumbent position; Average HUT, the average of the parameters during the whole time in the upright position.*

**TABLE 5:** Temporal changes in LF/HF in head-up tilt tests.

	**POTS *n* = 25**	***P*-value**	**Non-POTS *n* = 12**	***P*-value**
Change in LF/HF from Pre-Tilt supine to HUT 1 min	13.1 (7.0) to 15.4 (7.8)	<0.0001	13.4 (5.2) to 15.5 (5.6)	0.0005
Change in LF/HF from Pre-Tilt supine to HUT 2–4 min	13.1 (7.0) to 21.2 (10.6)	<0.0001	13.4 (5.2) to 19 (7.1)	0.0002
Change in LF/HF from Pre-Tilt supine to Average HUT	13.1 (7.0) to 34.4 (15.1)	<0.0001	13.4 (5.2) to 28.5 (12.3)	0.0032
Change in LF/HF from Pre-Tilt supine to HUT final	13.1 (7.0) to 44.4 (18.2)	<0.0001	13.4 (5.2) to 39.6 (19.8)	0.0059
Change in LF/HF from HUT 1 min to HUT final	15.4 (7.8) to 44.4 (18.2)	<0.0001	15.5 (5.6) to 39.6 (19.8)	0.0059
Change in LF/HF from HUT final to Post-Tilt supine	44.4 (18.2) to 21.8 (12.6)	<0.0001	39.6 (19.8) to 16.5 (7.7)	0.0020

*Values are means of the HRV parameters with standard deviation. P-values were computed with the sign test. POTS, subjects with postural tachycardia syndrome; non-POTS, subjects without postural tachycardia syndrome; HF, high-frequency components of heart rate variability spectra in ms^2^; LF,: low-frequency components of heart rate variability spectra in ms^2^; Pre-Tilt supine, 2 min before upright position in recumbent position; HUT 1 min, during the first minute of upright position; HUT 2–4 min, from the second to fourth minutes of upright position; HUT final, during the last minute of upright position; Post-Tilt supine, from the first to third minutes after returning to the recumbent position; Average HUT, the average of the parameters during the whole time in the upright position.*

**FIGURE 1:**
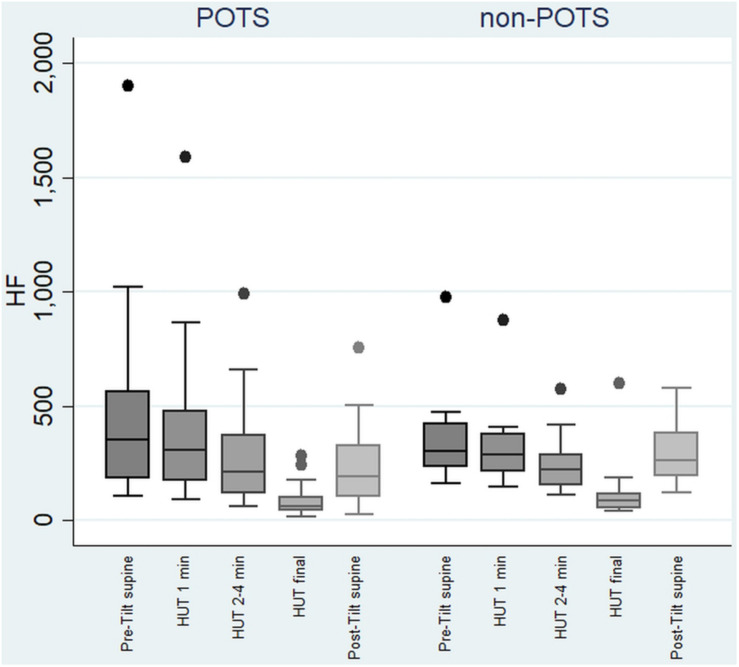
Temporal changes in HF during head-up tilt in subjects with and without postural tachycardia syndrome. The whiskers are adjacent lines, boxes are 25th to 75th percentile with median, and the dots are outliers. POTS, subjects with postural tachycardia syndrome; non-POTS, subjects without postural tachycardia syndrome; HF, high-frequency components of heart rate variability spectra in ms^2^; HUT, head-up tilt; Pre-Tilt supine, 2 min before upright position in recumbent position; HUT 1 min, during the first minute of upright position; HUT 2–4 min, from the second to fourth minutes of upright position; HUT final, during the last minute of upright position; Post-Tilt supine, from the first to third minutes after returning to the recumbent position.

In the beginning of the HUT test, the value of HF decreased in all the subjects in the POTS group and in the non-POTS groups compared with the value in recumbency. From the beginning of the upright position in the HUT tests, the value of HF gradually decreased toward the end of the upright position (*p* < 0.05) in both groups compared with the value in recumbency (see Figure 1). Compared with recumbency, in the first minute of the HUT test, the decrease in HF was significantly larger in the POTS group than in the non-POTS group (*p* = 0.04, *z* = 2.012). There was also a similar trend of bigger HF decrease in the POTS group throughout the whole HUT test, but the difference between the groups did not reach significance in other time intervals (data not shown).

**FIGURE 2:**
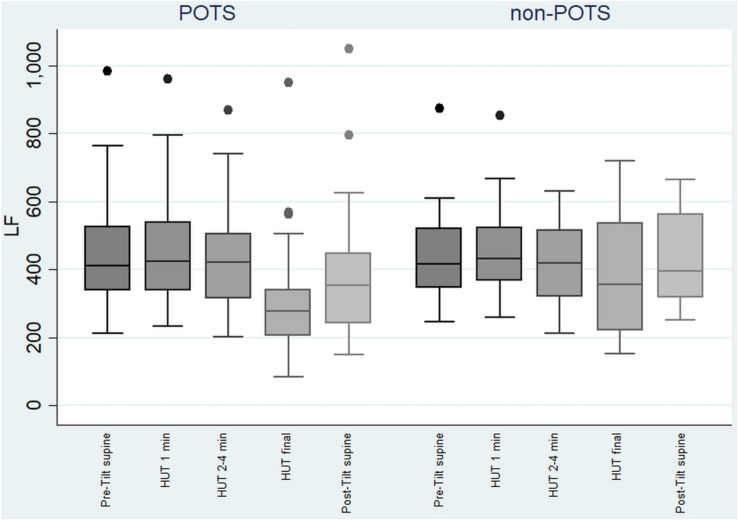
Temporal changes in LF during head-up tilt in subjects with and without postural tachycardia syndrome. The whiskers are adjacent lines, boxes are 25th to 75th percentile with median, and the dots are outliers. POTS, subjects with postural tachycardia syndrome; non-POTS, subjects without postural tachycardia syndrome; LF, low-frequency components of heart rate variability spectra in ms^2^; HUT, head-up tilt; Pre-Tilt supine, 2 min before upright position in recumbent position; HUT 1 min, during the first minute of upright position; HUT 2–4 min, from the second to fourth minutes of upright position; HUT final, during the last minute of upright position; Post-Tilt supine, from the first to third minutes after returning to the recumbent position.

**FIGURE 3:**
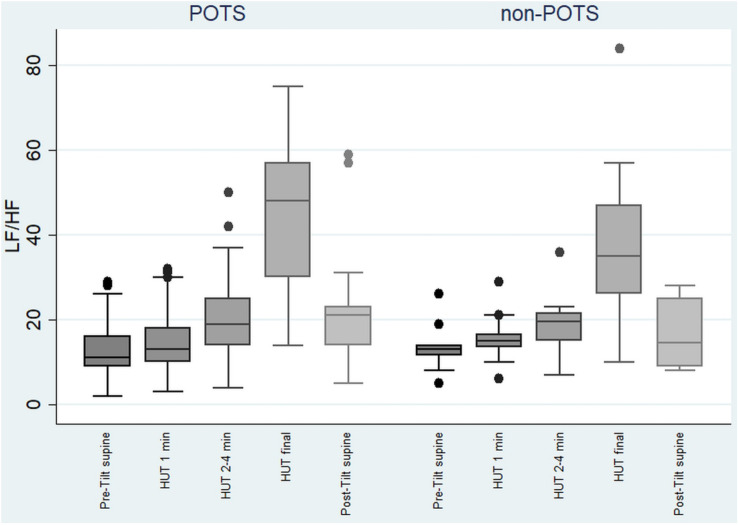
Temporal changes in LF/HF during head-up tilt in subjects with and without postural tachycardia syndrome. The whiskers are adjacent lines, boxes are 25th to 75th percentile with median, and the dots are outliers. POTS, subjects with postural tachycardia syndrome; non-POTS, subjects without postural tachycardia syndrome; HF, high-frequency components of heart rate variability spectra; LF, low-frequency components of heart rate variability spectra; LF/HF, LF/HF ratio in ms^2^; HUT, head-up tilt; Pre-Tilt supine, 2 min before upright position in recumbent position; HUT 1 min, during the first minute of upright position; HUT 2–4 min, from the second to fourth minutes of upright position; HUT final, during the last minute of upright position; Post-Tilt supine, from the first to third minutes after returning to the recumbent position.

The value of LF, at first, slightly increased and then gradually significantly decreased in the POTS group (*p* < 0.05), and in the non-POTS group the value of LF did not significantly increase in the beginning of the HUT or decrease toward the end (see Figure 2). In both groups, the value of LH/HF gradually increased toward the end of the upright position (*p* < 0.05) (see Figure 3), and the increase in LF/HF was significantly bigger in the POTS group in the first to fourth minutes from the beginning of the HUT test (*p* = 0.01, *z* = 2.5), whereas in the other time intervals there was only a trend of steeper LF/HF increase that did not reach significance. There were no significant differences between the values of the absolute change in LF between the groups (data not shown).

By way of illustration, Supplementary Figures S1, S2 present the selected parameters of one subject in each group – POTS and non-POTS – during their HUT tests.

There were no significant differences in the averages of the absolute values in HRV variables or BP variables (LF, HF, LF/HF, DBP, SPB, and MAP) between the groups in any of the previously described time intervals (data not shown). The individual variability between patients in HRV parameters is very high, especially in the frequency domain (Nunan et al., 2010; Sammito and Bockelmann, 2016); thus, it is not presumable to have statistically significant differences in group-level comparisons in a small group of patients. As described previously, the comparison of individual changes in the absolute values tells more. We did a power analysis *ex post facto* and it turned out that we would have needed a notably bigger study group (*n* = 162 in each group) to reach 0.05 statistical significance with 80% power in absolute difference of Pre-Tilt HF between the groups, for example.

There was no significant orthostatic hypotension reaction in any of the subjects (see Table 2).

## Discussion

The parasympathetic tone, i.e., HF, decreased in the HUT test in all tested adolescents, and the magnitude of the vagal (parasympathetic) attenuation gradually increased during the whole time upright. At the beginning of HUT, the parasympathetic tone attenuated significantly more in the POTS group. LF, in turn, increased significantly only in the POTS group in the beginning of HUT. Unexpectedly, the LF started to decrease toward the end of HUT in the POTS group, whereas this decrease was not seen in the subjects without POTS. LF/HF increased significantly in both groups in upright position, but the increase was steeper in the POTS group at the beginning.

Our results are in line with earlier studies, where patients with POTS had a significantly enhanced increase in LF/HF (Furlan et al., 1998), and a greater decrease in HF in the upright position period of HUT, compared with controls (Furlan et al., 1998; Corkal et al., 2014). Contrary to our findings, LF was increased at 3–5 min of the HUT test compared with recumbency in one study (Corkal et al., 2014). Previously, the stimulation of sympathetic tone during HUT, measured by microneurography, was lower in POTS than in controls, although resting muscle sympathetic nerve activity in POTS patients was higher than in controls (Furlan et al., 1998). The significant vagal attenuation we saw during HUT in both groups was also seen in another previous study (Baker et al., 2018a). In our study, there were no significant differences between the absolute sympathetic or parasympathetic HRV parameters between the groups before, during, or after HUT. Previously, there have been reports of lower parasympathetic and sympathetic tone in POTS patients than in controls before HUT in recumbency, and during the HUT tests (Stewart, 2000; Baker et al., 2018a). However, in a meta-analysis there were no significant differences in HF, LF, or LF/HF in recumbency after HUT in POTS patients compared with controls (Swai et al., 2019).

Our findings suggest that the central autonomic control might be impaired in POTS – because POTS patients have intolerable symptoms standing up – as there was a greater parasympathetic attenuation compared with the controls in the beginning of the standing and a greater attenuation in LF compared with the controls when standing for longer periods of time. This attenuation in HF and LF in the upright position could be associated with the symptoms of orthostatic intolerance in POTS patients. Much of the evidence in literature indicates that POTS may be a restricted autonomic neuropathy and our findings may support this conclusion.

To obtain functional standing hemodynamic, the ANS should increase sympathetic activity and decrease parasympathetic activity to increase the systemic vascular resistance and the cardiac output. For POTS patients, the overemphasized increase in HR may be a result of decreased sympathetic activity peripherally. The pathophysiology of POTS includes impaired sympathetically mediated vasoconstriction, but also excessive sympathetic drive, volume dysregulation, and deconditioning (Benarroch, 2012). On the other hand, it is possible that bed rest or deconditioning, often associated with POTS, in itself primarily affects the reflex controls of peripheral sympathetic activity (Benarroch, 2012).

Very high female to male ratios, such as 4:1, have been described in POTS (Sheldon et al., 2015). In our study, only a slight majority, 56%, of POTS patients were female, as in a previously published Finnish incidence study (Skufca et al., 2017). It could be that POTS is more common among Finnish males than in other countries for an as yet unknown reason as is indicated in both studies on the subject among the Finnish population. To our knowledge, there is no prevalence studies conducted on the kindred nations nor in the neighboring countries excluding Denmark. In the Danish population, the female/male ratio was approximately 3:1 (Brinth et al., 2018).

The autoimmune hypothesis of POTS etiology could be a link between immune triggers, such as viral and bacterial infections, or vaccinations, as patients often identify a triggering factor with the beginning of symptoms. Presence of other autoimmune disorders has been reported in 20% of POTS patients (Blitshteyn, 2015).

No associations between POTS and vaccines (Pandemrix, Cervarix, or NIP of Finland) or previous infections were found in our group. Of note, exceptionally few had been vaccinated with Cervarix in the non-POTS group, causing a statistical trend to higher coverage of the vaccine in the POTS group, though.

A higher proportion of POTS patients reported symptoms of antecedent infections in our group of adolescents (60%) than previously described for adults (35%) (Kimpinski et al., 2012). There was a slight trend to higher prevalence of previous *M. pneumoniae* infections in our POTS group than in the group without POTS, but the association was not significant. To our knowledge, no sign of this possible association has been published before.

In our study, the adolescents with POTS were sleepier than those without POTS, as described also in an earlier study (Mallien et al., 2014). In our group of POTS subjects, one adolescent had narcolepsy type 1, which is not a common comorbidity with POTS (Kim et al., 2018). We did not exclude any disease groups from our analysis because we wanted to do a systematic research of all the HUT tests done to the selected age group. What is more, the overall quality of life and perceived general health were alarmingly low in subjects with POTS in our group as previously shown (Benrud-Larson et al., 2002), and some of our subjects with POTS even used a wheelchair because of orthostatic intolerance. Still, most of them did not feel depressed. In a previous study of adolescent patients, up to 85% reported abdominal pain (Ojha et al., 2011), whereas the prevalence of abdominal pain was only 36% in our group.

### Strengths

Our study is original because, as far as we know, only a few studies of adolescents with POTS describe detailed demographic and history data, and HRV in HUT tests have been published.

### Limitations

The study sample in this age group was very small, hence large numerical differences in the parameters did not reach statistical significance. This probably predisposes to type II errors. Further, all the subjects in both groups had undergone the tests clinically; thus, we did not have an asymptomatic control group, and it might be that the subjects in the non-POTS group could have other forms of orthostatic intolerance that presents with sympathetic and vagal disturbances. This, on the other hand, creates strength to the research setup as the adolescents in both groups were equally symptomatic and the existence of POTS was the only thing that separated the groups.

We used the criteria recommended by Sheldon and coworkers (Sheldon et al., 2015), but even stricter diagnostic criteria for adolescent POTS have been introduced (Singer et al., 2012).

Blood pressure during HUT was obtained using PTT estimation. Even though individual calibration reduces the error associated with PTT, changes in autonomic state can affect the blood pressure values as much as 10 mmHg (Sklarsky et al., 2019).

All the results were controlled by sex and BMI, but we did not obtain information about the subjects’ physical activity nor socioeconomic status. This information would have been of value in finding possible confounding factors.

## Conclusion

Our findings indicate that parasympathetic tone attenuated in both groups in HUT, and the attenuation was bigger in POTS. In the beginning of HUT, LF increased more in POTS, but in the end of the upright position period, an attenuation in LF was seen in the POTS group. These results imply that the autonomic response to the upright position might be impaired in POTS correlating with orthostatic intolerance. There were no associations of previous infections or vaccinations with POTS. Subjects with POTS were significantly sleepier than subjects without POTS, and their quality of life was low.

## Data Availability Statement

The data analyzed in this study is subject to the following licenses/restrictions: Anonymized data will be shared by request from any qualified investigator. Requests to access these datasets should be directed to MO, maija.orjatsalo@helsinki.fi.

## Ethics Statement

The Institutional Review Board of the Vitalmed Helsinki Sleep Clinic approved the study. Written informed consent from the participants’ legal guardian/next of kin was not required to participate in this study in accordance with the national legislation and the institutional requirements.

## Author Contributions

MO has done the acquisition and analysis of the data and drafted the manuscript. All authors have made substantial contributions to conception and design of the manuscript and the interpretation of data. All authors have revised the manuscript for important intellectual content. All authors have seen and approved the manuscript and are accountable for all aspects of the work.

## Conflict of Interest

The authors declare that the research was conducted in the absence of any commercial or financial relationships that could be construed as a potential conflict of interest.

## Supplementary Material

FIGURE S1 Head-up tilt of a subject with postural tachycardia syndrome. Signals from above: HR, LF, HF, and LF/HF. HR, heart rate; HF, high-frequency components of heart rate variability spectra; LF, low-frequency components of heart rate variability spectra. The time interval between two vertical lines is 1 min.

FIGURE S2 Head-up tilt of a subject without postural tachycardia syndrome. Signals from above: HR, LF, HF, and LF/HF. HR, heart rate; HF, high-frequency components of heart rate variability spectra; LF, low-frequency components of heart rate variability spectra. The time interval between two vertical lines is 1 min. The red colored line is a 90 beats per minute indicator line.

TABLE S1 History of confirmed microbial infections and vaccinations prior to symptoms.

## Abbreviations

ADHD: attention-deficit hyperactivity disorder
ANS: autonomic nervous system
BMI: body mass index
BP: blood pressure
DBP: diastolic blood pressure
EBV: Epstein–Barr virus
ESS: Epworth Sleepiness Scale
HF: high-frequency components of the heart rate variability spectra
HHV6: human herpesvirus 6
HPV: human papillomavirus
HR: heart rate
HRV: heart rate variability
HUT: head-up tilt
LF: low-frequency components of the heart rate variability spectra
LF/HF: LF/HF ratio
MAP: mean arterial pressure
NIP: Finnish National Immunization Program
POTS: postural tachycardia syndrome
RDS: Rimon’s Brief Depression Scale
SBP: systolic blood pressure
WHO-5: World Health Organization–Five Well-Being Index.
