# Supplementary figures and images for: Heart Rate Variability in Head-Up Tilt Tests in Adolescent Postural Tachycardia Syndrome Patients
Source: Front Neurosci. 2020 Aug 11;14:725. doi: 10.3389/fnins.2020.00725 (PMC7432293; doi:10.3389/fnins.2020.00725)

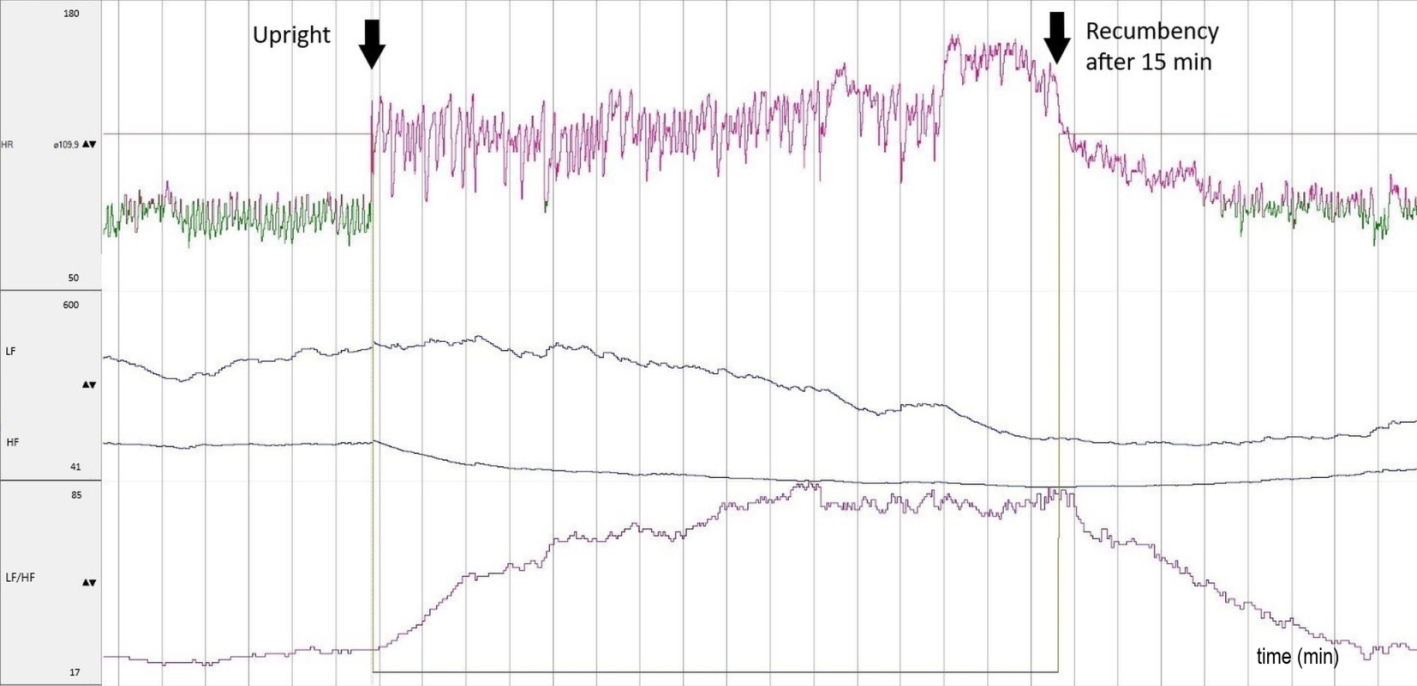

Supplement: FIGURE S1 — Head-up tilt of a subject with postural tachycardia syndrome. Signals from above: HR, LF, HF, and LF/HF. HR, heart rate; HF, high-frequency components of heart rate variability spectra; LF, low-frequency components of heart rate variability spectra. The time interval between two vertical lines is 1 min. [file Image_1.jpeg]

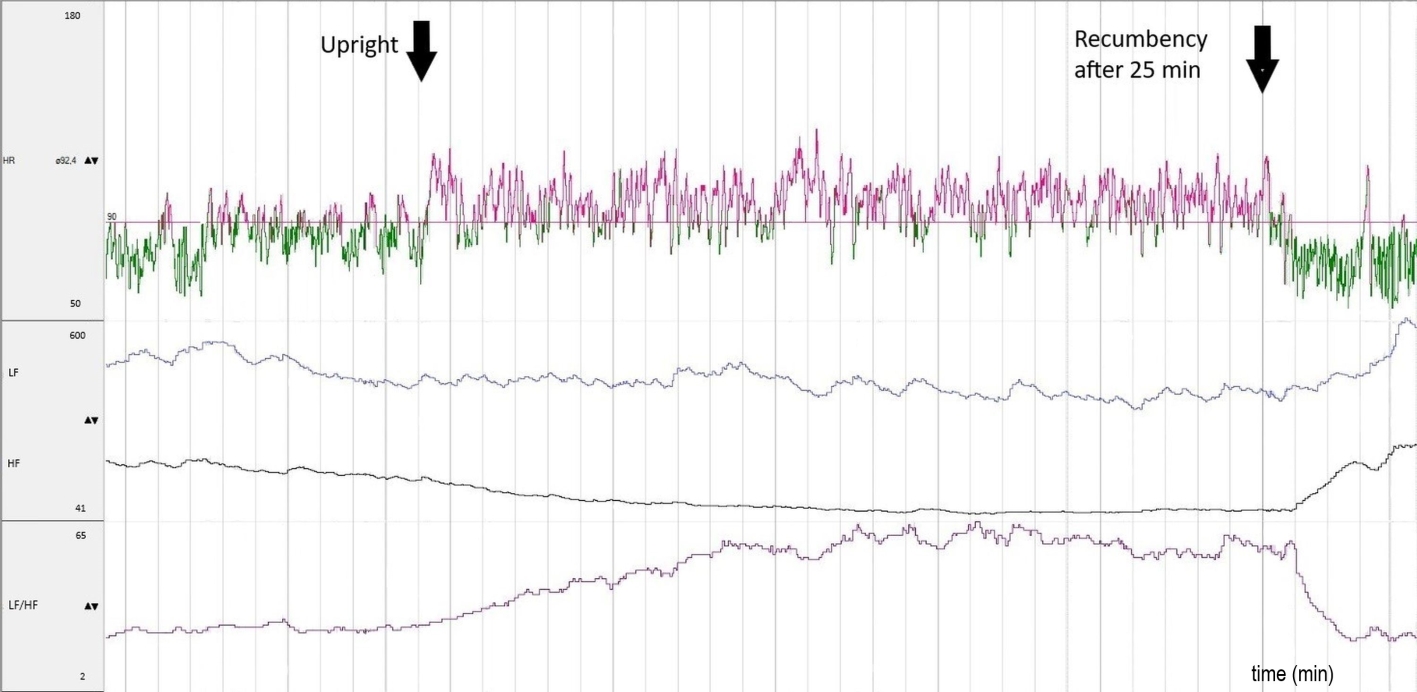

Supplement: FIGURE S2 — Head-up tilt of a subject without postural tachycardia syndrome. Signals from above: HR, LF, HF, and LF/HF. HR, heart rate; HF, high-frequency components of heart rate variability spectra; LF, low-frequency components of heart rate variability spectra. The time interval between two vertical lines is 1 min. The red colored line is a 90 beats per minute indicator line. [file Image_2.jpeg]
