# Supplementary material for: Heart Rate Variability in Head-Up Tilt Tests in Adolescent Postural Tachycardia Syndrome Patients
Source: Front Neurosci. 2020 Aug 11;14:725. doi: 10.3389/fnins.2020.00725 (PMC7432293; doi:10.3389/fnins.2020.00725)
Supplement: TABLE S1 — History of confirmed microbial infections and vaccinations prior to symptoms. [file Table_1.docx]

Table S1. History of confirmed microbial infections and vaccinations prior to symptoms.

|  | POTS n=25 | non-POTS n =12 | P |
| --- | --- | --- | --- |
| Streptococcal infection | 5/25 (20%) | 3/12 (25%) | 1.000 |
| HHV6 infection | 0/25 (0%) | 1/12 (8%) | 0.324 |
| EBV infection | 0/25 (0%) | 1/12 (8%) | 0.324 |
| Mycoplasma pneumoniae infection | 7/25 (28%) | 1/12 (8%) | 0.232 |
| Bordetella pertussis infection | 1/25 (4%) | 0/12 (0%) | 1.000 |
| Borrelia burgdorferi infection | 1/25 (4%) | 1/12 (8%) | 1.000 |
| Escherichia coli infection | 1/25 (4%) | 1/12 (8%) | 1.000 |
| Campylobacter jejuni infection | 1/25 (4%) | 0/12 (0%) | 1.000 |
| Chlamydia pneumoniae infection | 1/25 (4%) | 0/12 (0%) | 1.000 |
| Pandemrix vaccination in 2010 | 20/23 (87%) | 10/12 (83%) | 1.000 |
| Cervarix vaccination (HPV) | 7/23 (30%) | 1/11 (8%) | 0.216 |
| National vaccination program | 100% | 100% |  |

The values are numbers of subjects with percentage. P values were computed with Fisher’s exact test. POTS: subjects with postural orthostatic tachycardia; non-POTS: subjects without postural orthostatic tachycardia; HHV6: human herpesvirus 6; EBV: Epstein-Barr virus; HPV: human papillomavirus.
